# 中央型气道狭窄的诊断与腔内治疗

**DOI:** 10.3779/j.issn.1009-3419.2011.09.08

**Published:** 2011-09-20

**Authors:** 洪武 王, 浩波 张

**Affiliations:** 1 100028 北京，煤炭总医院肿瘤微创治疗中心 Minimal Invasive Tumor Therapy Center, Meitan General Hospital, Beijing 100028, China; 2 065400 香河，河北省香河县气管炎哮喘医院 Bronchitis & Bronchial Asthma Hospital of Xianghe County, Xianghe 065400, China

中央型气道狭窄（central airway stenosis, CAO）是指由于发生于气管、主支气管和右中间段支气管病变引起的狭窄^[1]^。由于其病因复杂，治疗方法多种多样，目前临床还缺乏统一的认识。近年来随着气管镜介入治疗方法的兴起，发表的文献也越来越多，但均缺乏统一的诊断和疗效判断标准。早在2007年，德国著名的气管镜介入治疗专家Freitag等^[2]^发表了18位欧美专家的共识，对CAO的定义、分类进行了详细解读，为该类疾病的研究奠定了基础。但这一方案并未引起世界各国专家的共鸣，国内也未见深入研究。作者根据多年来的工作经验，结合欧美专家的共识，提出了CAO统一的诊断和治疗意见，供国内同行参考。

## 1 病因及分类

CAO根据病因可分为良性和恶性。根据病变部位和性质又可分为功能性病变和结构性病变。功能性病变包括气管软化、复发性多发性软骨炎，结构性病变包括管内型、管壁型、管外型和混合型病变。管内型常见的病因^[3]^包括：①良性，如气管-支气管炎、气道内结核、白喉、梅毒等；气管内异物、结石；气管内良性病变，如气管淀粉样变、炎性肉芽肿、迷走的甲状腺等；气管内良性肿瘤，如纤维瘤、脂肪瘤、平滑肌瘤、错构瘤等。②恶性，气管内原发恶性肿瘤，如鳞癌、腺癌、腺样囊性癌、肉瘤、恶性淋巴瘤等；气管内转移瘤，常来自于肺、食道、肾、甲状腺等。管壁型常见的病因包括：①良性，如气管支气管软化症、气道烧伤、气管术后、放疗后及插管后所致的气道瘢痕狭窄等。在国外，肺移植、结节病、Wegener肉芽肿、支气管淀粉样变等疾病则占有较大比例。②恶性，如气道内原发恶性肿瘤（鳞癌、腺癌、腺样囊性癌、肉瘤、恶性淋巴瘤等）和气道内转移癌（常来自于肺、食道、肾、甲状腺等）。管外型病因包括增大的甲状腺、胸腺、淋巴结以及食道异物和气道外肿瘤压迫等。混合型为各种类型混杂在一起，常见于原发或继发的恶性肿瘤。

## 2 诊断

根据患者的临床表现、CT及气管镜表现，CAO的诊断并不难。

### 2.1 临床表现

患者表现为不同程度的咳嗽、气喘、呼吸困难以及病变远端反复的下呼吸道感染。呼吸困难常以吸气性为主，活动后加重；患者痰液较多，但咳出费力，有发生窒息的危险。体检可闻及喘鸣音，伴有下呼吸道感染的可闻及湿啰音。为了准确地反映病情轻重，作者把临床常用的指标用符号代表其严重程度（表 1，表 2），并根据气道狭窄的程度进行分级（表 3）。

**1:** 临床症状及体征严重程度分级 Classification of clinical manifestations and signs

Items	Classification			
	No (-)	Slight (1^+^)	Moderate (2^+^)	Severe (3^+^)
Cough	No	Interrupt cough, normal life	Between slight and severe	Frequent cough, abnormal sleep and life
Sputum	No	Sputum volume < 5 mL/d	Sputum volume 6 mL/d-20 mL/d	Sputum volume > 20 mL/d
Gasp	No	Gasping when activity, normal life	Between slight and severe	Gasping when rest, abnormal sleep and life
Asthma	No	Seldom wheezing rale	Sporadic wheezing rales	Full wheezing rales

**2:** 气促评分^[4]^ Shortbreath score

Grade	Symptoms
0	Normal
1	Shortbreath when fast walking
2	Shortbreath when normal walking
3	Stop walking due to shortbreath when normal walking
4	Shortbreath after slight activity

**3:** 气道狭窄程度的分级^[2]^ Degree of airway stenoses

Degree	Decrease (%) in cross-sectional area
0	0
Ⅰ	≤25
Ⅱ	> 25, ≤50
Ⅲ	> 50, ≤75
Ⅳ	> 75, ≤90
Ⅴ	> 90

### 2.2 影像学表现

根据影像学表现，CAO可分为9种类型（图 1）。

**1:**
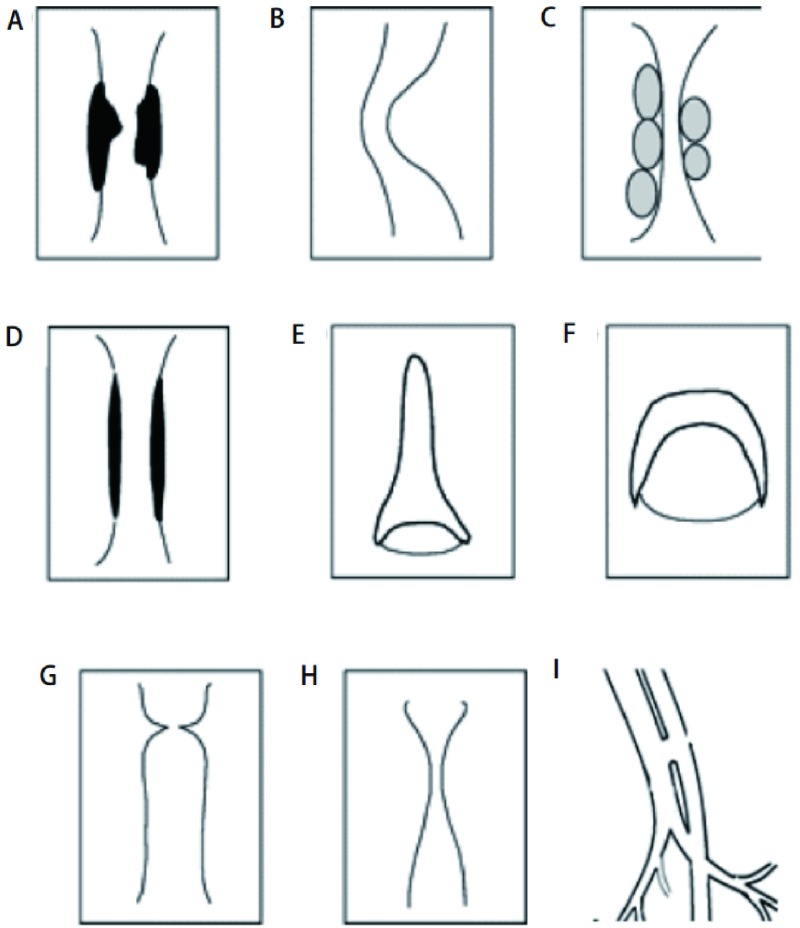
气道狭窄病变的模式图。A：腔内肿瘤或肉芽肿；B：扭曲或弯折；C：外压性狭窄；D：瘢痕性狭窄；E：剑鞘样气管；F：膜塌陷；G：蹼样狭窄；H：锥形狭窄（沙漏样狭窄）：I：气道瘘。 Schematic representations of the basic types of stenosis. A: intraluminar tumour or granulation; B: distortion or buckling; C: extrinsic compression; D: scar stricture; E: scabbard trachea; F: floppy membrane; G: abrupt transition (web stenosis); H: tapered transition (hourglass stenosis); I: airway fistulas.

### 2.3 气管镜表现

CAO的气管镜表现可分为9种类型（图 2）。9种类型中A、C、F、I多为恶性病变，其它均为良性病变。恶性病变中可简单地归纳为两大征象：（1）直接征象，即在气管镜下直接窥见肿瘤，是中央型肺癌在镜下的主要特征。根据其生长特性大致分为两种：①增生性改变，包括结节状、菜花状（桑椹样）、息肉状、乳头状等改变，有时癌肿表面覆盖乳白色坏死组织。癌肿常突向管腔，造成不同程度的阻塞。②浸润性改变，癌肿在支气管粘膜层或粘膜下层呈浸润状生长，可见到粘膜表面粗糙不平、局部增厚隆起、触之易出血、管腔呈不同程度不同形态的狭窄（如漏斗状、裂隙状、唇样等）或阻塞。（2）间接征象，即在支气管镜下未直接窥见明确的肿瘤体，为癌组织穿透支气管壁的外膜层向肺内生长。而管腔内仅表现为粘膜充血、水肿、糜烂、溃疡、增厚、僵硬、嵴增宽及管腔受压狭窄等非特异性改变。近年来荧光支气管镜和超声内镜的广泛应用为肺癌的早期诊断提供了更可靠的保证。

**2:**
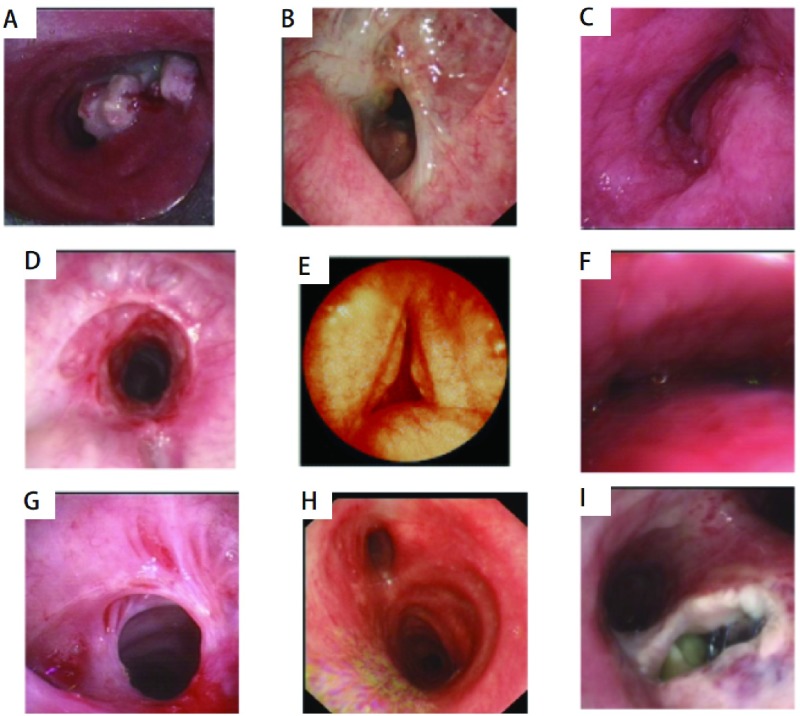
气管镜所见气道狭窄病变的形态（与模式图对应）。A：腔内肿瘤或肉芽肿；B：扭曲或弯折；C：外压性狭窄；D：瘢痕性狭窄；E：剑鞘样气管；F：膜塌陷；G：蹼样狭窄；H：锥形狭窄（沙漏样狭窄）；I：气管食管瘘。 Bronchoscopic imagings of the basic types of airway diseases. A: intraluminar tumour or granulation; B: distortion or buckling; C: extrinsic compression; D: scar stricture; E: scabbard trachea; F: floppy membrane; G: abrupt transition (web stenosis); H: tapered transition (hourglass stenosis); I: airway fistulas.

## 3 治疗

欧洲呼吸学会（European Respiratory Society, ERS）与美国胸科协会（American Thoracic Society, ATS）在“ERS/ATS Statement on interventaional pulmonology”中曾概括了气管镜介入治疗的主要技术，包括硬质支气管镜检术、经支气管针吸活检术、荧光支气管镜技术、支气管内超声、支气管镜介导下的激光、高频电灼、氩等离子体凝固（argon plasma coagulation, APC）、冷冻、气道内支架置入、支气管内近距离后装放疗、光动力治疗、气道内高压球囊扩张、支气管镜引导气管插管和氧气导管置入术等^[5]^。临床中可能需要将几种方法联合起来应用，因此必须熟悉各种方法的优缺点。

### 3.1 冷冻治疗

根据焦耳-汤姆逊（Joule-Thomson）原理，高压CO_2_气体通过小孔释放、节流膨胀制冷产生低温，最低温度可达-80 ℃，在冷冻探针的前段形成一定大小的冰球。将冰冻探头的金属头部放在组织表面或推进到组织内，使其在周围产生最大体积的冰球，在冷冻状态下将探头及其粘附的组织取出，此谓“冻取”。可以反复插入探头，直至将腔内的异常组织全部取出。如将冰冻探头的金属头部放在组织表面或推进到组织内，使其能在周围产生最大体积的冰球，持续冷冻1 min-3 min，复温后再进行另外2个冷冻-复温周期，移动探头，直至将所有能看到的组织全部冷冻，组织原位灭活，不必将冷冻组织取出，此谓“冻融”。

冻取主要用于气道内良、恶性病变组织、异物、坏死物质等，可在硬质镜或可弯曲性支气管镜（纤支镜或电子支气管镜）下进行。如采用硬质气管镜来实施冷冻治疗，操作在直视下进行，且简便、快捷、安全。经冷冻治疗后，患者的支气管阻塞症状可以很快减轻，生活质量得以改善。冻取后会有不同程度的出血，应结合APC或止血药止血。

近年来冻融治疗在良性气道狭窄的治疗中发挥越来越重要的作用，常用于创伤性气道狭窄、肉芽肿、气道结核等的治疗。气道瘢痕狭窄病变首先采取APC，再结合冻取，将管腔扩大，残留部位采用冻融^[6, 7]^。

已有研究^[2]^提示冷冻治疗和放射治疗具有协同作用。对于局限性的支气管癌，当不能手术治疗时常采用放射治疗，但用此法治疗的患者平均生存时间仅为20个月。只有35%的患者其局部肿瘤经放疗后消失。肿瘤阻塞引起肺不张患者，如果没有进行局部治疗而仅用放疗，只有21%的患者在放疗后肺可以复张。放射治疗在冷冻治疗后2周开始实施。

### 3.2 热消融治疗

包括激光、高频电刀（或APC）、微波等，能迅速减小肿瘤，畅通气道，缓解梗阻症状。对肿瘤较大，呼吸困难较明显者，应首选热消融治疗或冻切，先减轻管腔阻塞程度，然后配合放疗、光动力治疗和局部化疗等，必要时可配合气管内支架治疗。

各种热消融治疗的适应证相似，主要用于气道狭窄和出血的处理^[8, 9]^，但各种方法又有其优缺点。若组织与气道之间留有一定空隙、组织基底部较小或以蒂相连，可使用电圈套器套扎组织基底部，通电并缓慢收紧电圈套器直至组织切除；若组织较大、与气道之间空隙小且基底部较大，可使用电探针或电刀直接自组织表面或侧面由浅入深进行电烧或电切。若病变较弥漫或出血可用APC。但高频电刀是一种接触式烧灼，其电极前端易粘附坏死组织，需及时将坏死物清除。激光能量较高，但消融范围较小，且仪器昂贵，操作较难，易穿孔。微波设备便宜，操作简便，但效率较低。APC价格适中，为非接触式烧灼，效率较高，易于操作，目前在临床已广泛应用，但易着火，需谨慎操作。微波较激光、高频电刀、APC等更安全，对深层组织损伤小，穿孔、出血等并发症发生率极低。不足之处在于作用比较慢，每次操作耗时较长。

### 3.3 近距离放射治疗

腔内近距离放疗通常有两种方法。一种为腔内后装放疗，即先将盛有同位素的施源器或导源管送到合适的病变部位，经X线核实位置，再经治疗计划系统计算及优化剂量分布，获得满意结果后进行治疗。治疗结束后放射源可自动回到储源器内。后装近距离放射治疗的优点是患者可得到精确的治疗，且医务人员隔室遥控操作，非常安全。腔内近距离放疗一般与外放疗或腔内消融治疗结合应用。另外一种为放射粒子植入，通常是将放射性粒子捆绑在内支架上，既对狭窄的气管起支撑作用，又对肿瘤进行近距离放疗，控制肿瘤的进一步生长。亦可在支气管镜直视下将^125^I粒子直接植入到无法手术切除的大气管肿瘤、气道周围转移的淋巴结或肿瘤中，以解除大气管内肿瘤所致的气道堵塞和阻塞性肺炎等临床症状，肿瘤局部控制率可达85%^[10]^。

### 3.4 局部药物治疗

对明确为恶性气管内肿瘤患者，可配合冷冻、热疗，瘤体内注射化疗药，起到协同治疗作用。腔内注射常用的药物有化疗药（如顺铂、丝裂霉素、表阿霉素）、无水酒精、白介素-2（interleukin-2, IL-2）、基因药物（目前用于临床的药物有重组人p53腺病毒注射液，如今又生）等。近年来重组人p53腺病毒对中晚期头颈部鳞癌、肺癌采用瘤体内注射方式给药，取得非常好的疗效。

### 3.5 光动力治疗（photodynamic therapy, PDT）

PDT是先将光敏剂注入人体，光敏剂在进入机体后，会特异性地聚集于肿瘤部位并与肿瘤细胞结合，当用特定波长的激光照射后，会产生光化学反应（称为光敏反应），由此产生的光毒性物质会破坏肿瘤细胞和血管，从而抑制肿瘤生长^[11, 12]^。PDT疗法对早期气管-支气管癌可达根治效果，对晚期肿瘤则为姑息治疗手段。对于气管腔内较大的肿瘤采用光动力治疗前，可采用消融治疗祛除病灶，减少病灶厚度后再行PDT，常可提高疗效。

### 3.6 内支架置入

气道支架的绝对适应证是管外型气道狭窄、气道瘘和功能性气道狭窄（如气管软化、复发性多发性软骨炎）。管内型及管壁型气道狭窄则应以消融治疗为主，慎放支架。从气道狭窄的形态来看，腔内肿瘤或肉芽肿、瘢痕性狭窄、蹼样狭窄均不适合直接放置支架，而其它6种形态的病变可首选支架。不稳定的气道结核严禁放置任何支架，良性气道支架严禁放置永久性金属支架（无论是裸支架还是被膜支架）^[13, 14]^。隆突附近的病变如需放支架应首选分叉支架，特别是气道瘘应首选分叉型被膜金属支架封堵瘘口，必要时再同时放置食管支架^[15, 16]^。

### 3.7 球囊导管扩张^[17]^

无论是良性还是恶性近端气道狭窄，均可造成患者活动后胸闷、气急、呼吸困难以及反复发生肺部感染。采用支气管镜导入球囊导管，对狭窄的近端气道实施球囊扩张，可使狭窄部位的气道全周产生多处纵向小裂伤，裂伤处被纤维组织充填，从而达到狭窄部位扩张的目的。球囊导管扩张适应于各种原因引起的中心气道纤维性或非纤维性狭窄，包括：①创伤性气道狭窄，如肺移植或支气管肺癌肺叶切除术后吻合口狭窄、长期气管内插管或气管切开所致管腔狭窄、外伤、吸入毒性烟雾或烧伤、气管内介入治疗后及异物反应等所致管腔狭窄；②支气管内膜病变，如结节病、结核、支气管淀粉样变、韦格纳肉芽肿所致管腔狭窄；③先天性病变所致管腔狭窄；④良、恶性肿瘤所致气道狭窄。

球囊扩张术方法简单、安全、见效快，不需要全身麻醉，不需要特殊设备和复杂技术，可以避免激光治疗等所致的支气管穿孔，相对于外科手术和支架置入等其它方法更加经济、安全且创伤小，可作为各种病变所致的良性瘢痕性气管支气管狭窄的首选治疗。不足之处在于，为达到满意效果时常需反复进行。在置入支架前先对狭窄气道进行球囊扩张，可避免支架置入时支架置入器卡在狭窄处导致窒息，并且扩张后可选用较大的支架避免移位。单纯进行球囊扩张而不置入支架，气道容易再狭窄，如与冷冻治疗结合应用可大大降低复发率。

无论良性气道狭窄还是恶性气道狭窄，单一治疗方法很难达到理想治疗效果，需多种方法联合应用。对瘢痕性气道狭窄可首选球囊导管扩张联合冷冻治疗；如气道狭窄严重，可先选用热消融治疗将管腔扩大，再结合球囊导管扩张或冷冻治疗。对肉芽肿性或恶性肿瘤病变，应先选择冻取、电圈套器或其它消融治疗，将阻塞的病变清除，再结合冻融、药物注射等治疗，必要时选用支架置入。对轻度气道狭窄，在局麻下应用软镜即可进行介入治疗，而对严重气道狭窄或病情较重的患者，宜在全麻下插入硬质镜、气管插管或喉罩等进行，以减轻患者痛苦，减少气管镜介入治疗过程中的风险。Wang^[18]^曾报道194例大气道狭窄患者共接受了334次硬质镜检查，平均每例患者接受1.6次操作。气管内及支气管内狭窄分别采用电圈套器、冷冻、APC等综合治疗措施。气道内肿瘤包括原发肿瘤76例，转移性肿瘤69例。良性狭窄最常见病因为瘢痕狭窄，其次为良性肿瘤、原发性肉芽组织增生、异物、气管软化和复发性多发性软骨炎。硬质镜首次治疗后气道狭窄程度均明显下降，其中支气管的下降程度要大于主气管。首次治疗后KPS明显升高，气促评分明显下降。

总之，CAO无论发生于成人还是儿童都是很严重的疾病，临床上必须有统一的认识和规范的治疗方案。应从症状的描述、影像学及气管镜的表现等方面使用统一的分类标准以便于比较和研究，并且应根据病变部位和病变程度采取不同的治疗策略。
